# *Caenorhabditis inopinata* shows reduced attraction and increased head-swinging compared with *C. elegans* in plate chemotaxis

**DOI:** 10.17912/micropub.biology.001977

**Published:** 2026-01-15

**Authors:** Eddy Sukmawinata, Melis Konno, Xiaolan Li, Masaya Ono, Taisei Kikuchi

**Affiliations:** 1 Parasite Systems Biology, Department of Integrated Biosciences, Graduate School of Frontier Sciences, The University of Tokyo, Tokyo, Tokyo, Japan; 2 Research Centre for Veterinary Science, Research Organization for Health , National Research and Innovation Agency, Jakarta, Jakarta, Indonesia; 3 Faculty of Medicine, University of Miyazaki, Miyazaki, Miyazaki, Japan

## Abstract

Chemoreception underpins essential animal behaviors. *Caenorhabditis inopinata* , a close relative of *C. elegans* that inhabits fig syconia, provides an opportunity to test how microhabitat shapes odor preference. Using two-point chemotaxis assays, we compared these species across six volatile odorants. *C. elegans* showed strong attraction to all odorants, whereas *C. inopinata* responded only to 2,4,5-trimethylthiazole, 2-butanone, and diacetyl. In addition, *C. inopinata* moved more slowly under both odorant and non-odorant conditions, displayed frequent head-swinging, and rarely executed pirouette-like turns. These findings indicate divergence in odor preference and locomotory behavior, suggesting differences in chemotactic navigation associated with distinct ecological contexts.

**Figure 1. Chemotaxis to six odorants and tracking patterns of C. inopinata and C. elegans:**
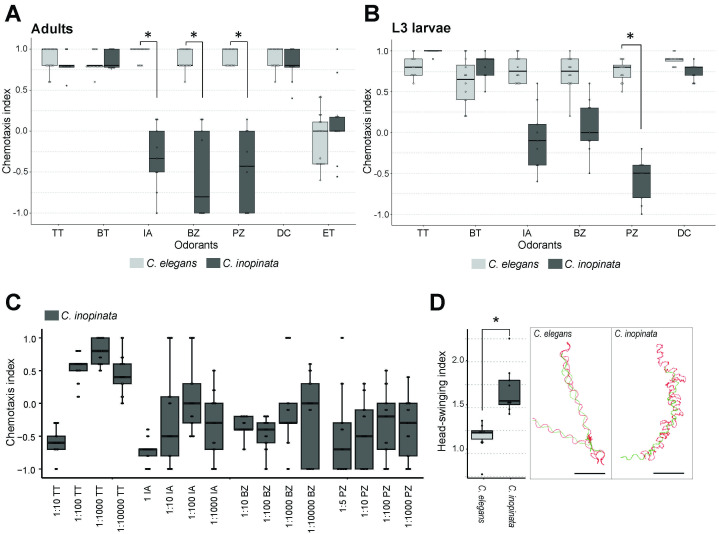
Figure 1. Chemotaxis to six odorants and tracking patterns of *C. inopinata* and *C. elegans* .Two-point plate chemotaxis was conducted for adults **(A)** and L3 larvae **(B)** . A fresh 5-cm NGM plate was used for each trial. The odorant and ethanol control were placed on opposite sides of the plate, and their left–right positions were alternated across trials. The chemotaxis index (CI) was calculated as CI=(B−A)/(A+B), where A and B are the numbers of animals at the control and odorant scoring area, respectively. Boxplots show the distribution of per-plate chemotaxis indices; central lines indicate medians, boxes the interquartile range, whiskers the 1.5× IQR, and circles the individual plate values. Odorants: TT, 2,4,5-trimethylthiazole; BT, 2-butanone; IA, isoamyl alcohol; BZ, benzaldehyde; PZ, pyrazine; DC, diacetyl; ET, ethanol only. *C. elegans* showed robust attraction to all odorants, whereas *C. inopinata* lacked attraction to BZ, IA, and PZ but responded to TT, BT, and DC. **(C)** Chemotaxis indices of *C. inopi* nata were measured across a dilution series of each odorant (1:10–1:10,000 in ethanol; higher ratios indicate lower concentrations). *C. inopinata* showed little or no attraction to BZ, IA, and PZ across dilutions, whereas attraction to TT, BT, and DC was restricted to higher concentrations. In contrast, *C. elegans* responded over a broader dilution range (not shown). **(D)** Boxplots of the head-swinging index (head-track length/centroid-track length) under odorant conditions for each species. Points are animals, boxes show IQR, center lines the median, whiskers 1.5× IQR. Representative tracks from single adults on odorant plates are shown at the right: green = centroid trajectory, red = head-tip trajectory. Scale bar=5 mm. Between-species or -condition differences are indicated by asterisks (P<0.05 in two-sided rank-based tests; see Statistics).

## Description

**Description**

Chemotaxis guides diverse nematode behaviors, including foraging, mating, danger avoidance and host association, and has been extensively characterized in *Caenorhabditis elegans* , where several odorants reliably elicit attraction (e.g., benzaldehyde, isoamyl alcohol, diacetyl) (Ward, 1973; Bargmann, 2006). *C. inopinata* is a close relative of *C. elegans* with distinct ecology and a reported reduction in its chemosensory GPCR repertoires (Kanzaki *et al.* , 2018), raising the question of whether its odor preferences match those of *C. elegans* . In this study, we investigated whether attraction to these odorants is conserved between *C. elegans* and *C. inopinata* .

We compared plate-based chemotaxis of *C. inopinata* (NKZ35) with *C. elegans* ( N2 ) using a two-point assay (odorant vs ethanol control) (Bargmann *et al.* , 1993; Margie *et al.* , 2013). Six commonly used *C. elegans* attractants were assayed: 2,4,5-trimethylthiazole (TMT), 2-butanone, diacetyl, benzaldehyde, isoamyl alcohol, and pyrazine. The chemotaxis index (CI) was defined as (B − A)/(A + B), where A and B are counts at control and odorant spots after a fixed assay period.

As expected, adult *C. elegans* showed robust attraction to all six odorants under our conditions ( Fig. 1A ). In contrast, adult *C. inopinata* displayed positive chemotaxis to TMT, 2-butanone, and diacetyl but little or no attraction to benzaldehyde, isoamyl alcohol, or pyrazine (CIs near zero or weakly negative across independent assays). This pattern was recapitulated at the L3 stage ( Fig. 1B ), indicating that the divergence is not limited to adults. Independent biological replicates, including a blinded repeat of the adult assays, confirmed reproducibility. No-odorant negative controls yielded CIs ~0 for both species, and odorant/control positions were alternated between plates to minimize spatial bias.

To test whether the difference was quantitative (sensitivity shift), we titrated benzaldehyde and pyrazine across concentrations known to attract *C. elegans* . *C. inopinata* remained weak or non-attracted across the tested range, whereas *C. elegans* showed the expected positive CIs ( Fig. 1C ). For 2,4,5-trimethylthiazole (TMT), both species produced a bell-shaped response with reduced attraction or repulsion at the highest dose, consistent with the *C. elegans* literature (Yoshida *et al.* , 2012). Taken together, these titrations are more consistent with a qualitative difference in odor preference than with a simple quantitative sensitivity shift, within the doses and stages examined.

During initial observations of worms on chemotaxis assay plates, we noticed clear differences in movement pattern between these species during chemotaxis assays. As *C. inopinata* locomotion has not been previously characterized, we first quantified its baseline movement parameters in the absence of odorants and found that *C. inopinata* moved significantly more slowly on the examination plates (Wilcoxon rank-sum test, p < 0.05, Extended Data Table S1) and showed larger-amplitude, higher-frequency lateral head excursions (head-swinging) than *C. elegans* . We then asked whether these differences persist in a chemotactic context by measuring the same parameters, including speed, under attractant-present conditions (1:1000 2,4,5-trimethylthiazole; Extended Data Table S1). Under these conditions, *C. inopinata* showed more pronounced head-swinging than *C. elegans* ( Fig. 1D ) and executed pirouette-like reorientations only very rarely, whereas pirouette events (Pierce-Shimomura *et al.* , 1999) were readily observed in *C. elegans* (Wilcoxon rank-sum test, p < 0.05, Extended Data Table S2). At the L3 stages pirouette-like movements were slightly more observable but still significantly less frequent in *C. inopinata* than in *C. elegans* (Extended Data Table S2). Together, these analyses provide the first quantitative description of *C. inopinata* locomotion and indicate that, across both control and chemotactic conditions, *C. inopinata* shows movement patterns characterized by pronounced head-swinging and infrequent pirouette-like reorientations.

We interpret these data as evidence for divergence in chemosensory preference between sister species rather than a general locomotor deficit in *C. inopinata* as *C. inopinata* shows robust attraction to three odorants under identical assay conditions. This behavioral divergence is consistent with ecological differences between the species and with genomic reports that *C. inopinata* has fewer chemosensory GPCRs than *C. elegans* (Kanzaki *et al.* , 2018). While the underlying mechanisms are beyond the scope of this study, our findings motivate straightforward follow-ups, including finer concentration–response sampling, simple adaptation time-courses, and tests of candidate GPCRs and neurons guided by existing annotations.

## Methods

**Materials and Methods**

**Strains and maintenance.** *C. elegans* N2 and *C. inopinata* NKZ35 were maintained on NGM seeded with *E. coli* OP50.1. Unless noted, *C. elegans* were kept at 20°C and *C. inopinata* at 25°C. Age-synchronised worms were obtained by hypochlorite treatment (Stiernagle, 2006). Adult *C. elegans* hermaphrodites and *C. inopinata* females used in the chemotaxis assays were collected within 8 hours after the first egg was observed on the plate.

**Chemotaxis assays.** Assays were performed on 5-cm NGM plates with two scoring spots (odorant vs ethanol control) containing sodium azide. Odorant diluted with ethanol (2 µl) was dropped onto the agar over one mark and 2 µl of ethanol was dropped over the other mark. Adults (≈10 per plate) were tracked for 60 min; L3 assays ran 120 min. Worms located in the left (A) or right (B) half of the plate were counted as control or odorant, respectively, whereas worms remaining in the central 1-cm-wide zone (C) were excluded from analysis. The chemotaxis index (CI) was calculated as (B – A)/(A + B). Biological replicates were obtained using independently prepared worms on at least three separate days. In addition, the results were confirmed by a second experimenter under blinded labels.

**Odorants.** Unless otherwise noted, odorants were diluted in ethanol to concentrations commonly used as attractants for adult *C. elegans* (e.g. (Bargmann *et al.* , 1993; Colbert and Bargmann, 1997; Choi *et al.* , 2018)): 2,4,5-trimethylthiazole (1:1,000; Sigma-Aldrich 219185), 2-butanone (1:10,000; Nacalai Tesque 22507-55), isoamyl alcohol (1:100; Tokyo Chemical Industry TCI-I0289), benzaldehyde (1:1,000; Nacalai Tesque; 04037-75), pyrazine (1:100; Sigma-Aldrich; P56003), and diacetyl (1:100; Tokyo Chemical Industry TCI-B0682).

**Tracking and movement measures.** Adult worms were recorded on 5-cm NGM plates with or without an attractant source (2,4,5-trimethylthiazole). Head and centroid positions were tracked using WormLab (MBF Bioscience, Williston, VT, USA). Instantaneous speed was calculated as the frame-to-frame centroid displacement divided by the frame interval; per-animal average speed was taken as the median of instantaneous speeds after excluding gaps and frames with immobility (<0.02 cm s⁻¹). Because the software can only accurately track worms moving at speeds up to 500 µm/s, individual data exceeding this threshold were treated as outliers and removed from the statical analysis. Track length was computed as the summed Euclidean distance across frames after excluding gaps. A head-swinging index was computed per animal as head&nbsp;track&nbsp;length/centroid&nbsp;track&nbsp;length. Index distributions were compared between species and between ethanol-only vs. attractant plates using rank-based tests as specified under *Statistics* . Pirouettes (reorientations) (Pierce-Shimomura *et al.* , 1999) were detected by WormLab with a criterion that the cumulative turning angle exceeded 120° within 3 s accompanied by a concurrent speed drop of ≥40% relative to the animal's 2-s pre-event baseline. Rates are reported as events min⁻¹.

**Statistics.** All statistical analyses were conducted in R. Pairwise comparisons between species for each odorant were performed using the Wilcoxon rank-sum test. Effect sizes (median differences) and their 95% confidence intervals were estimated by bootstrap resampling. Multi-group comparisons were assessed with the Kruskal–Wallis test followed by Dunn's post hoc test with Benjamini–Hochberg adjustment.

**Extended Data availability**

All raw plate counts, metadata (strain, stage, plate, odorant, concentration, assay time), and analysis scripts are deposited at Zenodo (https://doi.org/10.5281/zenodo.17846486) as Extended Data accompanying this article.

&nbsp;

## Data Availability

Description: Extended Data Tables. Resource Type: Dataset. DOI: https://doi.org/10.22002/f888j-90g44
